# Homology modeling and molecular docking of human pituitary adenylate cyclase-activating polypeptide I receptor

**DOI:** 10.3892/mmr.2014.2419

**Published:** 2014-07-24

**Authors:** LUSHENG WU, WENHUA GUANG, XIAOJIA CHEN, AN HONG

**Affiliations:** 1 Biomedicine Institute, College of Life Science and Technology, Jinan University, Guangzhou, Guangdong 510632, P.R. China; 2 Guangdong Provincial Key Laboratory of Bioengineering Medicine, Jinan University, Guangzhou, Guangdong 510632, P.R. China; 3 National Engineering Research Center of Genetic Medicine, Jinan University, Guangzhou, Guangdong 510632, P.R. China

**Keywords:** homology modeling, molecular docking, GPCR, PAC1R

## Abstract

Pituitary adenylate cyclase-activating peptide I receptor (PAC1R) is member of the B class of G protein-coupled seven-transmembrane receptors, with molecular functions associated with neural cell differentiation, regeneration and the inhibition of apoptosis. However, the integrity of the protein structure is difficult to be determined *in vitro*. In the present study, the physicochemical properties of PAC1R were analyzed, the extracellular, transmembrane and intracellular regions were constructed and a three-dimensional structure model of PAC1R was produced using extracellular loop region optimization and the energy minimization homology modeling method. Preliminary studies on the PAC1R protein and ligand interactions used a molecular docking method. The results indicated that the interaction sites of PAC1R were at Ile63, Ser100 and Gln105. These were the sites where the PAC1R combined with a hydrazide small molecule inhibitor. This study provides a theoretical basis for further studies on the model for the development of PAC1R target drugs.

## Introduction

Pituitary adenylate cyclase-activating polypeptide (PACAP) is a member of the vasoactive intestinal polypeptide/glucagon/growth hormone-releasing factor/secretin superfamily (1). This family of proteins has numerous physiological roles, including functions in the immune system, hormone secretion regulation, neurotrophic effects and neural restoration (2–7). PACAP can transfer extracellular signals into an intracellular signal, through the activation of the PAC1 receptor (PAC1R) (8). It has been previously shown that PACAP can induce the growth of the rabbit trigeminal ganglion and neurite outgrowth *in vitro*, and it has been verified further by an *in vitro* study that the intracellular signal can accelerate the recovery of corneal sensitivity (9). The development of PAC1R agonists as novel therapeutic agents may therefore show potential for promoting nerve regeneration. Research into the binding of ligands to PAC1R is required for developing specific PAC1R agonists.

**Figure 1:**
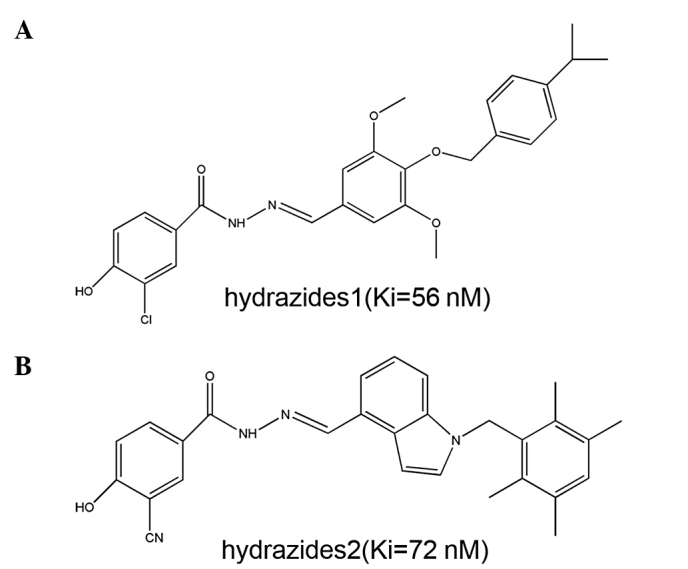
Molecular structure of hydrazides 1 and 2. The Ki of the combination of each small molecule hydrazide with the pituitary adenylate cyclase-activating polypeptide I receptor was (A) 56 and (B) 72 nm (13).

G protein-coupled receptors (GPCRs) are the drug target for numerous diseases. At present, ~50% of known drug targets are GPCRs (10). PAC1R is a member of the class B GPCRs. Sun *et al* (11) used nuclear magnetic resonance spectroscopy to analyze the crystal structure of PACAP (6–38) and the PAC1R extracellular domains (11). However, the study failed to analyze the structure of the whole PAC1R transmembrane domain. Furthermore, the interactive mode between PACAP38 and PAC1R has not been studied (12). A previous study used site-directed mutagenesis to investigate the influence of three amino acids of PACAP (6–38), Tyr10, Arg14 and Lys21, on the binding of PACAP (6–38) to the extracellular domains of PAC1R (11). Beebe *et al* (13) reported the molecular structure of two small-molecule inhibitors, Hydrazides1 and Hydrazides2 (Fig. 1), and their interaction with PAC1R. The binding strength IC50 value between the small-molecule inhibitor and the receptor was subsequently determined.

The present study aimed to predict and analyze the physicochemical properties of the PAC1R protein, according to the amino acid sequence of PAC1R. The transmembrane, intracellular and extracellular spatial structures of the PAC1R protein were constructed using Discovery Studio 2.5 (DS2.5; Accelrys Software Inc., San Diego, CA, USA) software with a homology-modeling module. The key binding sites and regions of PAC1R were preliminarily studied by molecular docking methodology in order to provide theoretical support for the study of the binding mode between ligands and PAC1R, and for the further development of PAC1R agonists.

## Materials and methods

### Materials

The primary sequence of PAC1R protein was obtained from the Swiss-Prot database (available at http://www.uniprot.org/), and the homology template was obtained through retrieving the protein database (http://www.rcsb.org/pdb/home/home.do).

### Bioinformatics analysis of the amino acid sequence

The physicochemical properties of PAC1R molecules were analyzed with the ProtParam tool (http://web.expasy.org/protparam/). Hydrophobicity analysis was performed for the primary sequence of PAC1R with the Kyte and Doolittle algorithm within the ProtScale online software (http://www.expasy.ch/tools/protscale.html). The greater the positive value, the stronger the hydrophobicity, and vice versa. Where the hydrophobic value was between −0.5 and +0.5, the amino acid was considered to exhibit both hydrophilic and hydrophobic characteristics (14)

### Homology modeling and evaluation

The primary sequence of PAC1R was obtained from the Swiss-Prot website (P41586-3) (15). The PAC1R extracellular domains (ID: 2JOD and 3N94) and the seven transmembrane regions (ID: 2KS9) were identified through the Swiss-Model Alignment Mode tool on the Swiss-Model website for homology search (http://swissmodel.expasy.org/). Homology modeling was performed using the modeler module of the DS2.5 molecular simulation software. The Blosum-62 matrix was used with a gap penalty of 10 and a gap extension penalty of one. The modeler module of DS2.5 was used to build the three-dimensional models of PAC1R. The ‘number of models’ parameter was set to 100 and the remaining parameters were set to the default values. The best-fit models of the PAC1R were selected on the basis of the analysis results of the internal scoring function of Modeler, the Profile-3D program and the PROCHECK procedure. A suitable model was then selected for further energy optimization and analysis (16).

### Molecular docking

The PAC1R spatial structure obtained following the optimization of homology modeling was used as the initial conformation of the receptor protein. The appropriate module of the DS2.5 molecular simulation software was applied for preprocessing the Protein Data Bank file, including hydrogenation. The molecular structures of the PAC1R inhibitors Hydrazides1 and Hydrazides2 were drawn with DS2.5, and stored as a structure-data file following energy optimization. The spatial structures of the protein and small molecules were displayed using Pymol 1.5 software (Schrödinger LLC, Portland, OR, USA).

## Results

### Physicochemical property analysis for the PAC1R sequence Composition and physicochemical properties

**Figure 2:**
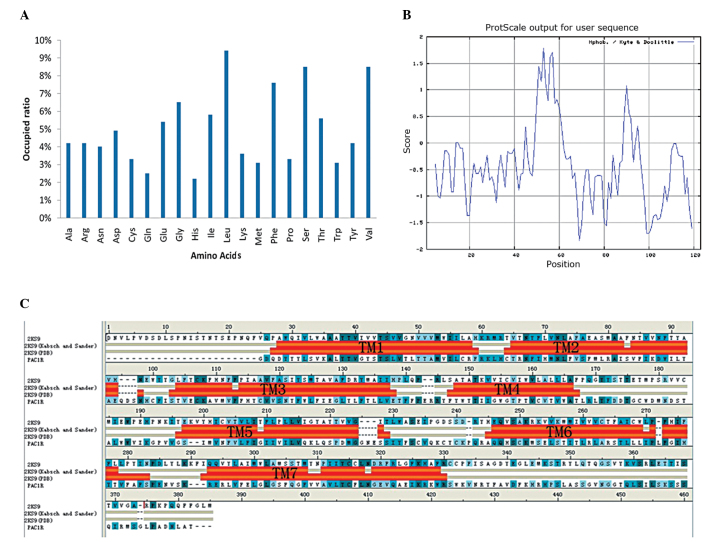
Alignment results between the PAC1R transmembrane and 2KS9 sequences and the amino acid sequence analysis. (A) Amino acid composition of the PAC1R protein (ID: P41586-3). The content of Leu, Val, Ser and Phe was high, whereas the content of His and Gln was low. (B) Prediction of hydrophilic/hydrophobic properties of the PAC1R (Kyte and Doolittle algorithm). There were eight evident hydrophobic domains in the protein, among which seven were the transmembrane domain of the protein. (C) Alignment results between the PAC1R transmembrane sequence and the 2KS9 sequence. TM indicates the transmembrane region of the PAC1R sequence and the orange regions represent the amino acid transmembrane regions of 2KS9 and PAC1R. PAC1R, pituitary adenylate cyclase-activating polypeptide I receptor.

The human PAC1R protein sequence was selected from the Swiss-Prot database (ID: P41586-3, removing the signal peptide from the front 20 amino acids prior to homology modeling). The physicochemical properties of the PAC1R protein were predicted using the ProtParam online tool: i) The molecular weight of the PAC1R protein was 49073.8 Da; ii) the theoretical isoelectric point was 6.07; iii) the atom composition was C_2,255_H_3,383_N_561_O_612_S_29_; iv) the molar digestion coefficient was 106,185 at 280 nm; v) the instability index was 47.45; vi) the fat coefficient was 88.95 and vii) the total average hydrophilicity was 0.183. The amino acid composition of PAC1R is shown in Fig. 2A, in which the amino acid residues with a positive charge accounted for 35% and the amino acid residues with a negative charge accounted for 40%. The content of Leu, Val, Ser and Phe was high, whereas that of His and Gln was low.

### Hydrophobicity analysis

The hydrophilicity and hydrophobicity of the PAC1R protein sequence were analyzed using the ProtScale online tool provided by Expasy (Swiss Institute of Bioinformatics), and the distribution of hydrophilic and hydrophobic amino acids of the protein was identified, as well a prediction of the secondary structure, including the transmembrane helices and the distribution of the amino acids on the surface of the protein. The maximum hydrophobicity of the protein was 3.6 and the minimum hydrophobicity was −2.4 (Fig. 2B). It was observed that there were eight evident hydrophobic domains in the protein, among which seven were the transmembrane domains of the protein.

### Homology modeling of the PAC1R sequence

The homology modeling of PAC1R was performed using DS Modeling 1.2. Similarity searches for the sequence were conducted using Position-Specific Iterative Basic Local Alignment Search Tool (PSI-BLAST) provided by the National Center for Biotechnology Information and, following the selection of the NCBI-BLAST contrast results, the sequence was labeled as 2JOD and 3N94. The A-chain homology of the extracellular domain and 2JOD and 3N94 was 99% (E-value=5×10^−69^) and 92% (E-value=1×10^−66^) respectively; however, there was no available (high homology) spatial structure template in the transmembrane domain and intracellular region of PAC1R.

It has been reported in the literature that the parathyroid hormone receptor I (PTHR1) from the family of B class GPCRs has been built with the human β2-adrenergic G protein-coupled receptor at a low matching ratio identity (8.5%; similarity, 25.5%) in the transmembrane domain as the module through segmented homology (17,18). However, since GPCRs have seven highly conservative spiral transmembrane domains and each domain has one to two conservative residues with conservative spatial structure, the complete spatial structure of PTH1R transmembrane domain was successfully constructed with the dual-template method. The PAC1R transmembrane and intracellular regional sequences were uploaded to the Swiss-Model website, and the template search contrast was then conducted using the Swiss-Model Alignment Mode. The results showed that the sequences had the highest sequence homology with the A-chain of 2KS9 (same rate, 15%; similar to the rate, 35.7%). Sequence alignment between the sequence and 2KS9 was performed and both the primary and secondary structures were analyzed using Align123 in DS Modeling 1.1, followed by a manual modification based on the hydrophobicity analysis result for sequence alignment. The final alignment was carefully evaluated and evidenced to be a match for the conserved residue data for the class B GPCR (19). The predicted transmembrane helices of the transmembrane protein module were analyzed and edited with DS2.5. The prediction identified that the transmembrane domain of the PAC1R was overlapping with the transmembrane domain of 2KS9 (Fig. 2C). During the homology modeling process for the construction of homology models using DS2.5, the transmembrane domain factor was added. The number of models was set as 100, the refine loops parameter was set as true and 200 models were constructed, whilst the remaining parameters were set to default.

**Figure 3:**
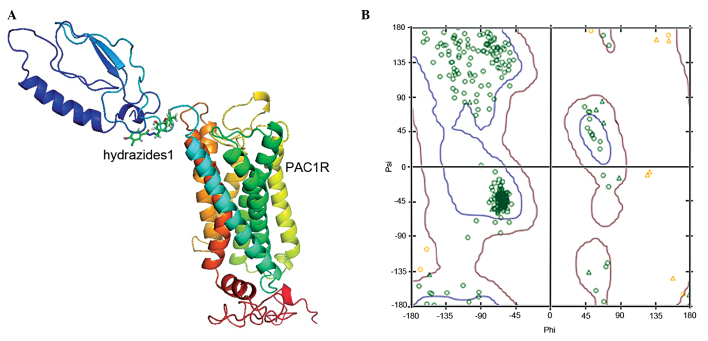
PAC1R homology modeling and molecular docking. (A) Spatial structure of PAC1R protein interaction with hydrazides1. Graphics were produced using the Pymol 1.5 software. (B) Ramachandran plot for the optimal model of PAC1R structure. The blue areas indicate the most suitable zone, the red areas indicate the acceptable zone, the areas outside of the red areas represent the unsuitable zone. PAC1R, pituitary adenylate cyclase-activating polypeptide I receptor.

The probability density function energy of the PAC1R model was checked, indicating a valid structure for the entire model, and the Ramachandran plots for local backbone conformation of each residue in the final models were produced by PROCHECK. The three-dimensional structural compatibility principle of the protein constructed was evaluated using the Profile-3D module of DS2.5 (20), and the average score was 144.02, between the maximum of 194.85 and minimum of 87.68. The stereochemical features of the model were constructed, including the evaluation of the stereochemical stability of the main and side chains, which was conducted using the Ramachandran module of DS2.5 software. A total of ≥90% of the ϕ and ψ stereochemistry in the main chain was distributed within the allowed region, and was in accordance with the principles of stereochemistry, suggesting that the model (Fig. 3A) was theoretically reliable. The Ramachandran plot, generated by the DS2.5 software, is shown in Fig. 3B; ~98.1% of the amino acids of the optimal model selected were within the allowed region (red region), suggesting that the model constructed may reflect the three-dimensional structure of the protein.

### Small-molecule inhibitor and PAC1R binding

**Figure 4:**
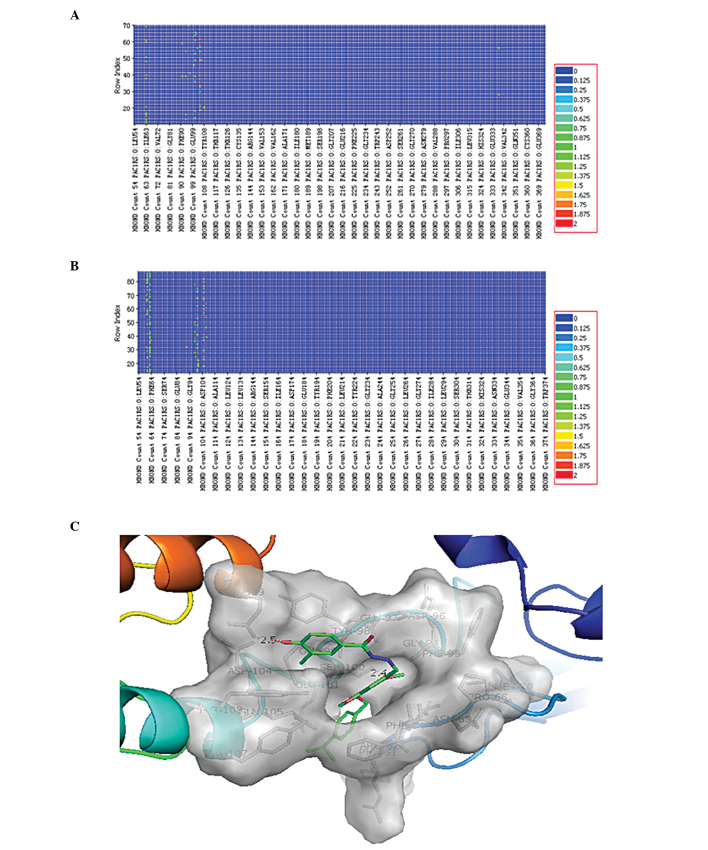
Binding mode and hydrogen bonding heat map between hydrazides1 and PAC1R. (A and B) The ordinate row index shows the different docking conformations. The abscissa represents the different amino acids of the PAC1R and the different colors indicate the number of hydrogen bonds of the docking conformation of the small molecule (A, Hydrazides1; B, Hydrazides2) with the PAC1R. (C) Hydrazides1 and the Ser100 and Glu339 sites of the PAC1R formed hydrogen bonding interactions, with chain lengths of 2.4 and 2.5 Å, respectively. Graphics were produced using Pymol 1.5 software. PAC1R, pituitary adenylate cyclase-activating polypeptide I receptor.

The optimal spatial structure of PAC1R protein, obtained through homology modeling, was modified with the Clean Protein tool in the DS2.5 software package. Under physiological conditions (pH 7.0), the hydrogenation could guarantee the protonation processing, which would be the initial conformation of the acceptor molecule following further energy optimization. The ligand molecules, Hydrazides1 and Hydrazides2, were compiled and stored as sd files with DS molecular simulation software, and the energy optimization for small molecules in the CHARMM force field was conducted. The binding between the small molecules and the receptor was implemented using the LibDock program of the DS simulation software package, and the active site of binding was predicted by the DS software. According to the binding result, there were 76 conformations in the binding of Hydrazides1 and the receptor, while there were 89 conformations in the binding of Hydrazides2 and the receptor. The hydrogen bond formed between the two small molecules and the receptor docking conformation was calculated with the ‘Analyze Ligand Poses’ process analysis. The hydrogen bonding heat map is shown in Fig. 4A and B. It can be observed that the Ile63, Ser100 and Gln105 residues of PAC1R formed a hydrogen bond with most Hydrazides1 and Hydrazides2 docking conformations. The conformation with the highest LibDock score for the docking of Hydrazides1 with the receptor was selected for the display (Fig. 4C). According to the docking results, in the selected conformation, Hydrazides1 formed hydrogen-bond interactions at the Ser100 and Glu339 of PAC1R, and the chain lengths were 2.4 and 2.5 Å respectively.

## Discussion

PACAP performs numerous biological functions, including promotion of nerve regeneration, neuroprotection, prevention of arteriosclerosis and regulation of energy metabolism (21–24). Following the binding of PACAP to its receptor, the extracellular signal is transferred into the cell, generating a biological response. The complete spatial structure of the seven-transmembrane receptors has not been analyzed, which has restricted the research and development of drugs targeting the PAC1R (25).

The first high-resolution template that was suitable for homology modeling of GPCRs became available in the year 2000 with the crystallization of bovine rhodopsin (26,27). The three-dimensional model of PTHR1 and the octopamine receptor 2 were constructed through two steps of homology modeling methods (17–19). The transmembrane regions exhibited low matching rates with 2RH1 as a template; however, the GPCR had the seven highly conserved, spiral transmembrane regions, and each transmembrane region had 1–2 conserved residues and the spatial structure was additionally conserved (17,27,28). Therefore, the three-dimensional model of the PAC1R was constructed by employing two-mode plate methods.

According to previous research (29), one of the active modes between the GPCR small-molecule inhibitors and the receptor took the extracellular domain of the receptor as the target, such as the binding model of small-molecule T-0632 with glucagon-like peptide 1 receptor (29). The Ki values of the small molecules Hydrazides1 and Hydrazides2 with the extracellular domain of the PAC1R protein, were 56 and 72 nM respectively (as shown in Fig. 1), with strong binding capability. A series of docking conformations were generated with the molecule docking method, and the binding conformation of the two molecules and the receptor was subsequently analyzed. The heat map of the hydrogen bonds generated in the receptor molecule is shown in Fig. 4. When analyzing the heat map of the hydrogen bonds, the key amino acids involved in the binding between the small molecules Hydrazides1 and Hydrazides2 and the receptor structure were directly observed (as shown in Fig. 4A and B). As observed from the position of the binding of Hydrazides1 and Hydrazides2 to the receptor (Fig. 3A), it can be predicted that these small-molecule inhibitors can prevent the normal interactions between the ligand agonist in the extracellular domain and the active region formed by the seven transmembrane regions of PAC1R through the free activity in the extracellular domain, thus achieving the inhibitory effect. In addition, according to the literature, the key domain of the binding ligand PACAP38 of PAC1R comprises residues 116–120, in the extracellular domain (11). It was suggested by the heat map of the hydrogen bonds that the hydrogen bond was formed between the two small-molecule inhibitors Hydrazides1 and Hydrazides2 and the Ser100 of PAC1R (the first 20 amino acids were the signal peptide and was removed, and the site Ser100 was Ser120). Each small-molecule inhibitor produces a steric hindrance in the binding of PACAP to PAC1R, which can inhibit the biological activity of PACAP competitively. The manner in which PACAP can bind with the PAC1R, the changes in the C-backbone of PAC1R, as well as how the extracellular signal is transferred into the cell through structural changes remains to be studied using methods such as molecular dynamics, isothermal titration calorimetry or surface plasmon resonance.
